# Analysis of stability for stochastic delay integro-differential equations

**DOI:** 10.1186/s13660-018-1702-2

**Published:** 2018-05-11

**Authors:** Yu Zhang, Longsuo Li

**Affiliations:** 1 Harbin University of Commerce School of Economics, Harbin, China; 2 Department of Mathematics, Harbin Institute of Technology, Harbin, China

**Keywords:** Stochastic delay integro-differential equations, Euler–Maruyama method, Split-step backward Euler method, Mean-square stability

## Abstract

In this paper, we concern stability of numerical methods applied to stochastic delay integro-differential equations. For linear stochastic delay integro-differential equations, it is shown that the mean-square stability is derived by the split-step backward Euler method without any restriction on step-size, while the Euler–Maruyama method could reproduce the mean-square stability under a step-size constraint. We also confirm the mean-square stability of the split-step backward Euler method for nonlinear stochastic delay integro-differential equations. The numerical experiments further verify the theoretical results.

## Introduction

Stochastic delay integro-differential equations, as the mathematical model, widely apply in biology, physics, economics and finance [1, 2]. Because of the stochastic delay integro-differential equations themselves, it is not easy to obtain an explicit solution for these kinds of equations, so it is necessary to research the numerical methods for numerical solution of stochastic delay integro-differential equations [3, 4]. Stability is the basic and important property of numerical methods for stochastic systems.

There are few results on the numerical methods to stochastic delay integro-differential equations. Ding et al. [5] dealt with the stability of the semi-implicit Euler method for linear stochastic delay integro-differential equations. Rathinasamy and Balachandran [6] proved mean-square stability of the Milstein method for linear stochastic delay integro-differential equations with Markovian switching under suitable conditions on the integral term. The condition under which the split-step backward Euler method was mean-square stable has been obtained by Tan and Wang [7, 8]. Rathinasamy and Balachandran [9] also analyzed *T*-stability of the split-step-*θ*-methods for linear stochastic delay integro-differential equations. Wu [10] investigated the mean-square stability for stochastic delay integro-differential equations by the strong balanced methods and the weak balanced methods with sufficiently small step-size. Numerical researches for stochastic delay integro-differential equations are not perfect enough. Therefore, it is extremely essential to develop the stability of the numerical methods to stochastic equations.

The paper is organized as follows. In Sect. 2 we will introduce related symbols and definitions. Some suitable conditions will be given to guarantee stability of the Euler–Maruyama method for stochastic delay integro-differential equations in Sect. 3. In Sect. 4, the split-step backward Euler method will be used to prove general mean-square stability of numerical solutions. In Sect. 5, we will discuss stability of nonlinear stochastic delay integro-differential equations. Furthermore, numerical experiments are provided in Sect. 6.

## Preliminaries

Throughout this paper, unless otherwise specified, let $(\Omega,\mathcal {F},P)$ be a complete probability space with a filtration $(\mathcal {F}_{t})_{t\geq0}$, which satisfies the usual conditions (i.e., it is increasing and right continuous while $\mathcal{F}_{0}$ contains all *P*-null sets). Let $|\cdot|$ be the Euclidean norm, $W(t)$ is Wiener process defined on the probability space, which be $\mathcal {F}_{t}$-adapted and independent of $\mathcal{F}_{0}$. Let $\tau>0$ and $C([-\tau,0];\mathbb{R})$ denote the family of all continuous $\mathbb {R}$-valued functions on $[-\tau,0]$, $C([-\tau,0];\mathbb{R}^{d})$ denote the family of all continuous functions *ξ* from $[-\tau,0]$ to $\mathbb{R}^{d}$, $\|\xi\|$ is defined by $\|\xi\|=\sup_{-\tau\leq t\leq 0}|\xi(t)|$. We assume $\xi(t)$, $t\in[-\tau,0]$ is the initial function, which is $\mathcal{F}_{0}$-measurable and right continuous, $E\|\xi\| ^{2}<\infty$. Let $\mathcal{C}_{\mathcal{F}_{0}}^{b}([-\tau,0];\mathbb{R})$ be the family of all $\mathcal{F}_{0}$-measurable bounded $C([-\tau,0];\mathbb {R})$-valued random variables $\xi=\{\xi(\theta): -\tau\leq\theta\leq0\}$.

As a matter of convenience, we first consider the following form of linear stochastic delay integro-differential equations:

1 $$ \left\{ \textstyle\begin{array}{l} dx(t)=[\alpha x(t)+\beta x(t- \tau)+\gamma \int_{t-\tau }^{t}x(s)\,ds]\,dt \\ \phantom{dx(t)=} +[\lambda x(t)+\mu x(t-\tau)+\eta \int_{t-\tau}^{t}x(s)\,ds]\,dW(t), \quad t\geq 0, \\ x(t)=\xi(t),\quad t\in[-\tau,0], \end{array}\displaystyle \right . $$

where $\xi(t)$ is initial function, and $\xi(t)\in C([-\tau,0];\mathbb {R})$, $\alpha, \beta, \gamma, \lambda, \mu, \eta\in\mathbb{R}$, $W(t)$ is a standard one-dimensional Wiener process and *τ* is the delay term.

Under the above assumptions, Eq. (1) has a unique solution $x(t)$. In order to analyze mean-square stability of two numerical methods, we introduce the following lemma [11].

### Lemma 2.1

*If*

2 $$ \alpha+|\beta|+|\gamma|\tau+\frac{1}{2}\bigl(|\lambda|+|\mu|+| \eta|\tau\bigr)^{2}< 0 $$

*the solution of Eq*. (1) *is said to be mean*-*square stable*, *that is*,

3 $$ \lim_{t\rightarrow\infty}E\big|x(t)\big|^{2}=0. $$

## Mean-square stability of the Euler–Maruyama method

Now, the Euler–Maruyama method applied to Eq. (1) one gets

4 $$ X_{n+1}=X_{n}+(\alpha X_{n}+\beta X_{n-m}+\gamma\bar{X}_{n})h+(\lambda X_{n}+\mu X_{n-m}+\eta\bar{X}_{n})\triangle W_{n}, $$

where $\xi=X_{0}$, $X_{n}$ is an approximation to the analytical solution $x(t_{n})$,n which is $\mathcal{F}_{t_{n}}$-measurable, $h>0$ is the given step-size, which satisfies $h=\frac{\tau}{m}$ for a positive integer *m*, $t_{n}=nh$, $n=-m,\ldots,0$, and we get $X_{n}=\xi (t_{n})$ when $t_{n}\leq0$, $\triangle W_{n}=W(t_{n+1})-W(t_{n})$ are independent $N(0,h)$ distributed stochastic variables. $\bar{X_{n}}$ approaches the integral term, this paper will choose a composite trapezoidal rule as the tool of the disperse integral to solve this case. We have

$$\begin{aligned} \bar{X_{n}}=\frac{h}{2}X_{n-m}+h\sum _{k=1}^{m-1}X_{n-k}+\frac{h}{2}X_{n}. \end{aligned}$$

### Definition 3.1

If there exists a $h_{0}>0$, for every step-size $h\in(0,h_{0}]$ with $h=\frac{\tau}{m}$, such that the numerical approximation $\{X_{n}\}$ produced by the Euler–Maruyama method satisfies

5 $$ \lim_{n\rightarrow\infty}E|X_{n}|^{2}=0 $$

then the numerical method applied to Eq. (1) is said to be mean-square stable.

### Theorem 3.1

*Under the condition* (2), *let* $h_{0}=\max\{h_{1},h_{2}\}$, *for step*-*size* $h\in (0,h_{0}]$, *we have*

$$\begin{aligned} \lim_{n\rightarrow\infty}E|X_{n}|^{2}=0 \end{aligned}$$

*then the Euler–Maruyama method applied to Eq*. (1) *is mean*-*square stable*, *where*

$$\begin{gathered} h_{1}=-\frac{2\alpha+2|\beta|+2|\gamma|\tau+(|\lambda|+|\mu|+|\eta|\tau )^{2}}{(|\alpha|+|\beta|+|\gamma|\tau)^{2}}, \\ h_{2}=\min\biggl\{ -\frac{1}{\alpha},-\frac{2\alpha+2|\beta|+2|\gamma|\tau +(|\lambda|+|\mu|+|\eta|\tau)^{2}}{(\alpha+|\beta|+|\gamma|\tau)^{2}}\biggr\} . \end{gathered}$$

### Proof

From Eq. (4), we obtain

6 $$ \begin{aligned}[b] X_{n+1}={}&(1+\alpha h+\eta \triangle W_{n})X_{n}+(\beta h+\mu\triangle W_{n})X_{n-m} \\ &+(\gamma h+\eta\triangle W_{n})\bar{X}_{n}. \end{aligned} $$

Squaring both sides of Eq. (6), we have

$$\begin{aligned} X_{n+1}^{2}={}&(1+\alpha h+\eta\triangle W_{n})^{2}X_{n}^{2}+( \beta h+\mu\triangle W_{n})^{2}X_{n-m}^{2}+( \gamma h \\ &+\eta\triangle W_{n})^{2}\bar{X}_{n}^{2}+2(1+ \alpha h+\eta\triangle W_{n}) (\beta h+\mu\triangle W_{n})X_{n}X_{n-m} \\ &+2(1+\alpha h+\eta\triangle W_{n}) (\gamma h+\eta\triangle W_{n})X_{n}\bar {X}_{n} \\ &+2(\beta h+\mu\triangle W_{n}) (\gamma h+\eta\triangle W_{n})X_{n-m}\bar {X}_{n} \\ ={}&\bigl(1+\alpha^{2}h^{2}+\lambda^{2}\triangle W_{n}^{2}+2\alpha h+2\lambda\triangle W_{n}+2 \alpha\lambda h\triangle W_{n}\bigr)X_{n}^{2} \\ &+\bigl(\beta^{2}h^{2}+2\beta\mu h\triangle W_{n}+\mu^{2}\triangle W_{n}^{2} \bigr)X_{n-m}^{2}+\bigl(\gamma^{2}h^{2} \\ &+2\gamma\eta h\triangle W_{n}+\eta^{2}\triangle W_{n}^{2}\bigr)\bar{X}_{n}^{2}+2\bigl[ \beta h(1+\alpha h+\eta\triangle W_{n}) \\ &+\mu\triangle W_{n}(1+\alpha h+\eta\triangle W_{n}) \bigr]X_{n}X_{n-m}+2\bigl[\gamma h(1+\alpha h+\eta\triangle W_{n}) \\ &+\eta\triangle W_{n}(1+\alpha h+\eta\triangle W_{n}) \bigr]X_{n}\bar{X}_{n} \\ &+2(\beta h+\mu\triangle W_{n}) (\gamma h+\eta\triangle W_{n})X_{n-m}\bar{X}_{n}. \end{aligned}$$

It follows from $2ab\leq|ab|(x^{2}+y^{2})$, where $a,b\in\mathbb{R}$, $\tau =mh$, that

7 $$ \begin{aligned}[b] 2X_{n-m}\bar{X}_{n}&=2X_{n-m} \Biggl(\frac{h}{2}X_{n-m}+h\sum_{k=1}^{m-1}X_{n-k}+ \frac{h}{2}X_{n}\Biggr) \\ &=hX_{n-m}^{2}+2hX_{n-m}\sum _{k=1}^{m-1}X_{n-k}+hX_{n}X_{n-m} \\ &\leq hX_{n-m}^{2}+h(m-1)X_{n-m}^{2}+h\sum _{k=1}^{m-1}X_{n-k}^{2}+ \frac {h}{2}\bigl(X_{n}^{2}+X_{n-m}^{2} \bigr) \\ &\leq\tau X_{n-m}^{2}+\frac{h}{2}X_{n-m}^{2}+h \sum_{k=1}^{m-1}X_{n-k}^{2}+ \frac{h}{2}X_{n}^{2}. \end{aligned} $$

According to the inequality $(a_{1}+a_{2}+\cdots+a_{n})^{2}\leq n(a_{1}^{2}+a_{2}^{2}+\cdots+a_{n}^{2})$,

8 $$ \begin{aligned}[b] \bar{X}_{n}^{2}&=h^{2} \Biggl(\frac{1}{2}X_{n-m}+\sum_{k=1}^{m-1}X_{n-k}+ \frac {1}{2}X_{n}\Biggr)^{2} \\ &\leq h^{2}\Biggl(\frac{1}{4}X_{n-m}^{2}+(m-1) \sum_{k=1}^{m-1}X_{n-k}^{2}+ \frac {1}{4}X_{n}^{2}+\frac{1}{2} \Biggl[(m-1)X_{n-m}^{2} \\ &\quad+\sum_{k=1}^{m-1}X_{n-k}^{2} \Biggr]+\frac{1}{2}\bigl(X_{n}^{2}+X_{n-m}^{2} \bigr)+\frac {1}{2}\Biggl[(m-1)X_{n}^{2}+\sum _{k=1}^{m-1}X_{n-k}^{2}\Biggr]\Biggr) \\ &\leq\tau\Biggl(\frac{h}{2}X_{n-m}^{2}+h\sum _{k=1}^{m-1}X_{n-k}^{2}+ \frac{h}{2}X_{n}^{2}\Biggr). \end{aligned} $$

In a similar way

9 $$ 2X_{n}\bar{X}_{n}=\tau X_{n}^{2}+ \frac{h}{2}X_{n-m}^{2}+h\sum _{k=1}^{m-1}X_{n-k}^{2}+ \frac{h}{2}X_{n}^{2}. $$

We note that $E(\triangle W_{n})=0$, $E[(\triangle W_{n})^{2}]=h$, and $X_{n}, X_{n-1},\ldots,X_{n-m}$ are $\mathcal{F}_{t_{n}}$-measurable. Substituting (7), (8), (9) into the above equation and taking expectations,

$$\begin{aligned} EX_{n+1}^{2}\leq{}&\bigl(1+\alpha^{2}h^{2}+ \lambda^{2}h+2\alpha h\bigr)EX_{n}^{2}+\bigl(\beta ^{2}h^{2}+\mu^{2}h\bigr)EX_{n-m}^{2} \\ &+\bigl(\gamma^{2}h^{2}+\eta^{2}h\bigr)\tau E \Biggl(\frac{h}{2}X_{n-m}^{2}+h\sum _{k=1}^{m-1}X_{n-k}^{2}+ \frac{h}{2}X_{n}^{2}\Biggr) \\ &+\bigl[\big|(1+\alpha h)\beta h\big|+|\lambda\mu|h\bigr]\bigl(EX_{n}^{2}+EX_{n-m}^{2} \bigr) \\ &+\bigl[\big|(1+\alpha h)\gamma h\big|+|\lambda\eta|h\bigr]E\Biggl(\tau X_{n}^{2}+\frac {h}{2}X_{n-m}^{2} \\ &+h\sum_{k=1}^{m-1}X_{n-k}^{2}+ \frac{h}{2}X_{n}^{2}\Biggr)+\bigl(|\beta \gamma|h^{2}+|\mu \eta|h\bigr) \\ &\times E\Biggl(\tau X_{n-m}^{2}+\frac{h}{2}X_{n-m}^{2}+h \sum_{k=1}^{m-1}X_{n-k}^{2}+ \frac {h}{2}X_{n}^{2}\Biggr). \end{aligned}$$

Let $Y_{n}=E|X_{n}^{2}|$, we have

$$\begin{aligned} Y_{n+1}\leq PY_{n}+QY_{n-m}+R\max _{n-m\leq i\leq n}(Y_{i}), \end{aligned}$$

where

$$\begin{gathered}\begin{aligned} P={}&1+\alpha^{2}h^{2}+\lambda^{2}h+2\alpha h+\big|(1+ \alpha h)\beta h\big|+|\lambda\mu |h+|\lambda\eta|\tau h \\ &+\big|(1+\alpha h)\gamma\tau h\big|, \end{aligned}\\ Q=\beta^{2}h^{2}+\mu^{2}h+\big|(1+\alpha h)\beta h\big|+| \lambda\mu|h+|\beta\gamma |\tau h^{2}+|\mu\eta|\tau h, \\ R=\bigl(\gamma^{2}h^{2}+\eta^{2}h\bigr) \tau^{2}+\big|(1+\alpha h)\gamma\tau h\big|+|\lambda\eta |\tau h+|\beta\gamma| \tau h^{2}+|\mu\eta|\tau h. \end{gathered}$$

So

$$\begin{aligned} Y_{n+1}\leq(P+Q+R)\max\Bigl\{ Y_{n},Y_{n-m},\max _{n-m\leq i\leq n}(Y_{i})\Bigr\} . \end{aligned}$$

It is clear that $Y_{n}\rightarrow0$ ($n\rightarrow\infty$) if $P+Q+R<1$, namely

$$\begin{gathered} 1+\alpha^{2}h^{2}+\lambda^{2}h+2\alpha h+2\big|(1+ \alpha h)\beta h\big|+2|\lambda\mu |h+2|\lambda\eta|\tau h+\beta^{2}h^{2}+ \mu^{2}h \\ \quad{}+2\big|(1+\alpha h)\gamma\tau h\big|+2|\beta\gamma|\tau h^{2}+2|\mu\eta|\tau h+\gamma^{2}h^{2}+\eta^{2}h< 1. \end{gathered}$$

Hence let

$$\begin{gathered} h_{1}=-\frac{2\alpha+2|\beta|+2|\gamma|\tau+(|\lambda|+|\mu|+|\eta|\tau )^{2}}{(|\alpha|+|\beta|+|\gamma|\tau)^{2}}, \\ h_{2}=\min\biggl\{ -\frac{1}{\alpha},-\frac{2\alpha+2|\beta|+2|\gamma|\tau +(|\lambda|+|\mu|+|\eta|\tau)^{2}}{(\alpha+|\beta|+|\gamma|\tau)^{2}}\biggr\} . \end{gathered}$$

By the condition (2), we know that $h_{1}>0$, $h_{2}>0$. If $h_{0}\in(0,h_{1})$, we have

$$\begin{gathered} \bigl(\alpha^{2}+2|\alpha\beta|+2|\alpha\gamma|\tau+ \beta^{2}+2|\beta\gamma|\tau +\gamma^{2}\tau^{2} \bigr)h^{2} \\ \quad{}+\bigl(2\alpha+2|\beta|+2|\gamma|\tau+\bigl(|\lambda|+|\mu|+|\eta| \tau\bigr)^{2}\bigr)h< 0. \end{gathered}$$

On the other side, we address the case $1+\alpha h>0$. If $h_{0}\in(0,h_{2})$, we get

$$\begin{gathered} \bigl(\alpha^{2}+2\alpha|\beta|+2\alpha|\gamma|\tau+ \beta^{2}+2|\beta\gamma|\tau +\gamma^{2}\tau^{2} \bigr)h^{2} \\ \quad{}+\bigl(2\alpha+2|\beta|+2|\gamma|\tau+\bigl(|\lambda|+|\mu|+|\eta| \tau\bigr)^{2}\bigr)h< 0. \end{gathered}$$

Let $h_{0}\in\max\{h_{1},h_{2}\}$; when $h\in(0,h_{0}]$, $P+Q+R<1$ always holds, then

$$\begin{aligned} \lim_{n\rightarrow\infty}Y_{n}=\lim_{n\rightarrow\infty}E|X_{n}|^{2}=0 \end{aligned}$$

then the Euler–Maruyama method applied to Eq. (1) is mean-square stable. The theorem is completed. □

## General mean-square stability of the split-step backward Euler method

Using the split-step backward Euler method applied to Eq. (1), we construct the numerical scheme as follows:

10 $$ \left \{ \textstyle\begin{array}{l} X_{n}^{\ast}=X_{n}+ (\alpha X_{n}^{\ast}+\beta X_{n-m}+\gamma\bar {X}_{n})h, \\ X_{n+1}=X_{n}^{\ast}+(\lambda X_{n}^{\ast}+\mu X_{n-m}+\eta\bar{X}_{n} )\triangle W_{n}; \end{array}\displaystyle \right . $$

the relevant definitions are in Sect. 3, if $1-\alpha h\neq0$, we can get the sequences$\{X_{n}^{\ast},n\geq0\}$ and $\{X_{n},n\geq1\}$ via (10), when given $X_{n}=\xi(nh)$ for $n\in\{-m,-m+1,\ldots,0\}$.

### Definition 4.1

For every step-size $h=\frac{\tau}{m}$, if any application of the split-step backward Euler method to Eq. (1) generates a numerical approximation $\{X_{n}\}$ that satisfies

11 $$ \lim_{n\rightarrow\infty}E|X_{n}|^{2}=0 $$

then the numerical method applied to Eq. (1) is said to be general mean-square stable.

### Theorem 4.1

*Under the condition* (2), *assume* $1-\alpha h\neq0$, *the split*-*step backward Euler method applied to Eq*. (1) *is generally mean*-*square stable*.

### Proof

Assume $1-\alpha h\neq0$ and implying $\alpha<0$; we can see from (10) that

12 $$ X_{n+1}=\frac{1+\lambda\triangle W_{n}}{1-\alpha h}(X_{n}+\beta hX_{n-m}+\gamma h\bar{X}_{n})+(\mu X_{n-m}+\eta \bar{X}_{n})\triangle W_{n}. $$

Squaring both sides of Eq. (12),

$$\begin{aligned} X_{n+1}^{2}={}&\biggl(\frac{1+\lambda\triangle W_{n}}{1-\alpha h}\biggr)^{2} \bigl(X_{n}^{2}+\beta ^{2}h^{2}X_{n-m}^{2}+ \gamma^{2}h^{2}\bar{X}_{n}^{2}+2\beta hX_{n}X_{n-m}+2\gamma hX_{n}\bar{X}_{n} \\ &+2\beta\gamma h^{2}X_{n-m}\bar{X}_{n}\bigr)+ \bigl(\mu^{2}X_{n-m}^{2}+\eta^{2}\bar {X}_{n}^{2}+2\mu\eta X_{n-m}\bar{X}_{n} \bigr)\triangle W_{n}^{2} \\ &+2\frac{1+\lambda\triangle W_{n}}{1-\alpha h}(X_{n}+\beta hX_{n-m}+\gamma h \bar{X}_{n}) (\mu X_{n-m}+\eta\bar{X}_{n})\triangle W_{n}. \end{aligned}$$

According to $2abxy\leq|ab|(x^{2}+y^{2})$ and $E(\triangle W_{n})=0$, $E[(\triangle W_{n})^{2}]=h$, substituting (7), (8), (9) into the above equation and taking expectations

$$\begin{aligned} EX_{n+1}^{2}\leq{}&\frac{1+\lambda^{2}h}{(1-\alpha h)^{2}}\Biggl[EX_{n}^{2}+ \beta ^{2}h^{2}EX_{n-m}^{2}+ \gamma^{2}h^{2}\tau E\Biggl(\frac{h}{2}X_{n-m}^{2} \\ &+h\sum_{k=1}^{m-1}X_{n-k}^{2}+ \frac{h}{2}X_{n}^{2}\Biggr)+|\beta |h \bigl(EX_{n}^{2}+EX_{n-m}^{2}\bigr) \\ &+|\gamma|hE\Biggl(\tau X_{n}+\frac{h}{2}X_{n-m}^{2}+h \sum_{k=1}^{m-1}X_{n-k}^{2}+ \frac{h}{2}X_{n}^{2}\Biggr) \\ &+|\beta\gamma|h^{2}E\Biggl(\tau X_{n-m}+ \frac{h}{2}X_{n-m}^{2}+h\sum _{k=1}^{m-1}X_{n-k}^{2}+ \frac{h}{2}X_{n}^{2}\Biggr)\Biggr] \\ &+\mu^{2}hEX_{n-m}^{2}+\eta^{2}\tau hE \Biggl(\frac{h}{2}X_{n-m}^{2}+h\sum _{k=1}^{m-1}X_{n-k}^{2}+ \frac{h}{2}X_{n}^{2}\Biggr) \\ &+|\mu\eta|hE\Biggl(\tau X_{n-m}+\frac{h}{2}X_{n-m}^{2}+h \sum_{k=1}^{m-1}X_{n-k}^{2}+ \frac{h}{2}X_{n}^{2}\Biggr) \\ &+\frac{|\lambda\mu|h}{1-\alpha h}\bigl(EX_{n}^{2}+EX_{n-m}^{2} \bigr)+\frac{2|\beta \lambda\mu|h^{2}}{1-\alpha h}EX_{n-m}^{2} \\ &+\frac{|\gamma\lambda\mu|h^{2}}{1-\alpha h}E\Biggl(\tau X_{n-m}+\frac {h}{2}X_{n-m}^{2}+h \sum_{k=1}^{m-1}X_{n-k}^{2}+ \frac{h}{2}X_{n}^{2}\Biggr) \\ &+\frac{|\lambda\eta|h}{1-\alpha h}E\Biggl(\tau X_{n}+\frac{h}{2}X_{n-m}^{2}+h \sum_{k=1}^{m-1}X_{n-k}^{2}+ \frac{h}{2}X_{n}^{2}\Biggr) \\ &+\frac{|\beta\lambda\eta|h^{2}}{1-\alpha h}E\Biggl(\tau X_{n-m}+\frac {h}{2}X_{n-m}^{2}+h \sum_{k=1}^{m-1}X_{n-k}^{2}+ \frac{h}{2}X_{n}^{2}\Biggr) \\ &+\frac{2|\gamma\lambda\eta|h^{2}}{1-\alpha h}\tau E\Biggl(\frac {h}{2}X_{n-m}^{2}+h \sum_{k=1}^{m-1}X_{n-k}^{2}+ \frac{h}{2}X_{n}^{2}\Biggr), \end{aligned}$$

in particular

$$\begin{aligned} EX_{n+1}^{2}\leq PEX_{n}^{2}+QEX_{n-m}^{2}+R \max_{n-m\leq i\leq n}E\bigl(X_{i}^{2}\bigr), \end{aligned}$$

where

$$\begin{gathered} P=\frac{1+\lambda^{2}h}{(1-\alpha h)^{2}}\bigl(1+|\beta|h+|\gamma|h\tau\bigr)+\frac {|\lambda\eta|h\tau}{1-\alpha h}+ \frac{|\lambda\mu|h}{1-\alpha h}, \\ \begin{aligned} Q={}&\frac{1+\lambda^{2}h}{(1-\alpha h)^{2}}\bigl(\beta^{2}h^{2}+|\beta|h+|\beta \gamma |\tau h^{2}\bigr)+\mu^{2}h+|\mu\eta|\tau h \\ &+\frac{|\lambda\mu|h}{1-\alpha h}+\frac{|\beta\lambda\eta|\tau h^{2}}{1-\alpha h}+\frac{|\gamma\lambda\mu|\tau h^{2}}{1-\alpha h}+\frac {2|\beta\lambda\mu|h^{2}}{1-\alpha h}, \end{aligned}\\ \begin{aligned} R={}&\frac{1+\lambda^{2}h}{(1-\alpha h)^{2}}\bigl(\gamma^{2}h^{2} \tau^{2}+|\gamma|\tau h+|\beta\gamma|\tau h^{2}\bigr)+ \eta^{2}\tau^{2}h+|\mu\eta|\tau h \\ &+\frac{|\gamma\lambda\mu|\tau h^{2}}{1-\alpha h}+\frac{|\lambda\eta|\tau h}{1-\alpha h}+\frac{|\beta\lambda\eta|\tau h^{2}}{1-\alpha h}+\frac {2|\gamma\lambda\eta|h^{2}}{1-\alpha h} \tau^{2}. \end{aligned}\end{gathered}$$

Let $Y_{n}=E|X_{n}^{2}|$, the above inequality turns into

$$\begin{aligned} Y_{n+1}\leq(P+Q+R)\max\Bigl\{ Y_{n},Y_{n-m},\max _{n-m\leq i\leq n}Y_{i}\Bigr\} . \end{aligned}$$

We conclude that $Y_{n}\rightarrow0$ ($n\rightarrow\infty$), if $P+Q+R<1$, that is,

$$\begin{gathered} \frac{1+\lambda^{2}h}{(1-\alpha h)^{2}}\bigl(1+|\beta|h+2|\gamma|h\tau\bigr)^{2}+\frac {2|\lambda\mu|h}{1-\alpha h}+ \frac{2|\lambda\eta|\tau h}{1-\alpha h}+\frac{2|\beta\lambda\mu|h^{2}}{1-\alpha h} \\ \quad{}+\frac{2|\gamma\lambda\mu|\tau h^{2}}{1-\alpha h}+\frac{2|\beta\lambda\eta |\tau h^{2}}{1-\alpha h}+\frac{2|\gamma\lambda\eta|\tau^{2}h^{2}}{1-\alpha h}+\bigl(|\mu|+|\eta| \tau\bigr)^{2}h< 1. \end{gathered}$$

Hence, we have

$$\begin{gathered} \bigl[\bigl(|\beta\lambda|+|\gamma\lambda|\tau\bigr)-\alpha\bigl(|\mu|+|\eta|\tau \bigr) \bigr]^{2}h^{2}+\bigl[\bigl(|\beta|+|\gamma|\tau\bigr)^{2}- \alpha^{2} \\ \quad{}+\bigl(2|\lambda|+2|\mu|+2|\eta|\tau\bigr) \bigl(|\beta\lambda|+|\gamma\lambda|\tau -\alpha\bigl(|\mu|+|\eta|\tau\bigr)\bigr)\bigr]h \\ \quad{}+2\alpha+2|\beta|+2|\gamma|\tau+\bigl(|\lambda|+|\mu|+|\eta|\tau\bigr)^{2}< 0. \end{gathered}$$

Let

$$\begin{aligned} F(h)=Ah^{2}+Bh+2\alpha+2|\beta|+2|\gamma|\tau+\bigl(|\lambda|+|\mu|+|\eta| \tau\bigr)^{2}, \end{aligned}$$

where

$$\begin{gathered} A=\bigl[\bigl(|\beta\lambda|+|\gamma\lambda|\tau\bigr)-\alpha\bigl(|\mu|+|\eta|\tau\bigr) \bigr]^{2}, \\ B=\bigl(|\beta|+|\gamma|\tau\bigr)^{2}-\alpha^{2}+\bigl(2|\lambda|+2| \mu|+2|\eta|\tau \bigr) \bigl(|\beta\lambda|+|\gamma\lambda|\tau-\alpha\bigl(|\mu|+|\eta|\tau\bigr)\bigr), \\\begin{aligned} \triangle={}&\bigl[\bigl(|\beta|+|\gamma|\tau\bigr)^{2}-\alpha^{2}+\bigl(2| \lambda|+2|\mu|+2|\eta |\tau\bigr) \bigl(|\beta\lambda|+|\gamma\lambda|\tau-\bigl(|\mu|+| \eta|\tau\bigr)\bigr)\bigr]^{2} \\ &-4\bigl[\bigl(|\beta\lambda|+|\gamma\lambda|\tau\bigr)-\alpha\bigl(|\mu|+|\eta|\tau \bigr) \bigr]^{2}\bigl[2\alpha+2|\beta|+2|\gamma|\tau+\bigl(|\lambda|+|\mu|+|\eta| \tau\bigr)^{2}\bigr]. \end{aligned}\end{gathered}$$

It is easy to see $A>0$, Because of the nature of a quadratic function, we can see that $F(h)<0$ holds for any $0< h<1$, when $\frac{-B+\sqrt {\triangle}}{2A}\geq1$, and the split-step backward method Euler is general mean-square stable. This proves the theorem. □

## Mean-square stability of the split-step backward Euler method for nonlinear stochastic systems

In this section, we will discuss the mean-square stability of the split-step backward Euler method for nonlinear stochastic delay integro-differential equations. Considering the following nonlinear stochastic equation:

13 $$ \left\{ \textstyle\begin{array}{l} dx(t)=f(x(t),x(t-\tau), \int_{t-\tau}^{t}x(s)\,ds)\,dt \\ \phantom{dx(t)=} +g(x(t),x(t-\tau), \int_{t-\tau}^{t}x(s)\,ds)\,dW(t),\quad t\geq0, \\ x(t)=\xi(t),\quad t\in[-\tau,0], \end{array}\displaystyle \right . $$

$f:\mathbb{R}^{d}\times\mathbb{R}^{d}\times\mathbb{R}^{d}\rightarrow\mathbb {R}^{d}$, $g:\mathbb{R}^{d}\times\mathbb{R}^{d}\times\mathbb{R}^{d}\rightarrow \mathbb{R}^{d\times m}$, $\xi(t)\in C([-\tau,0];\mathbb{R}^{d})$, $W(t)$ is an *m*-dimensional Wiener process and *τ* is a delay term. If *f* and *g* are sufficiently smooth and satisfy the Lipschitz condition and the linear growth condition, Eq. (13) has a unique strong solution $x(t)$, $t\in[-\tau,\infty)$ and $x(t)$ is a measurable, sample-continuous and $\mathcal{F}_{t}$ adapted process [12, 13].

The split-step backward Euler method applied to Eq. (13) yields

14 $$ \left\{ \textstyle\begin{array}{l} X_{n}^{\ast}=X_{n}+f (X_{n}^{\ast},X_{n-m},\bar{X}_{n} )h, \\ X_{n+1}=X_{n}^{\ast}+g(X_{n}^{\ast},X_{n-m}, \bar{X}_{n})\triangle W_{n}; \end{array}\displaystyle \right . $$

$X_{n}$, $X_{n}^{\ast}$, $\bar{X}_{n}$, *h*, $\triangle W_{n}$ are defined in Sects. 3 and 4.

### Lemma 5.1

([14])

*If there exist constant* $a_{1}$, $a_{2}$, $a_{3}$, $b_{1}$, $b_{2}$, $b_{3}$, *for all* $x,u,v\in \mathbb{R}^{d}$, *we have*

15 $$\begin{aligned}& \bigl\langle x,f(x,0,0)\bigr\rangle \leq-a_{1}|x|^{2} , \end{aligned}$$

16 $$\begin{aligned}& \big|f(x,u,v)-f(x,0,0)\big|\leq a_{2}|u|+a_{3}|v| , \end{aligned}$$

17 $$\begin{aligned}& \big|g(x,u,v)\big|^{2}\leq b_{1}|x|^{2}+b_{2}|u|^{2}+b_{3}|v|^{2} . \end{aligned}$$

### Theorem 5.1

*Suppose that Lemma *5.1 *holds and let*

18 $$ -a_{1}+a_{2}+a_{3}\tau+ \frac{1}{2}\bigl(b_{1}+b_{2}+b_{3} \tau^{2}\bigr)< 0. $$

*If there exists a* $h_{0}>0$, *for every step*-*size* $h\in(0,h_{0}]$, *we have*

$$\begin{aligned} \lim_{n\rightarrow\infty}E|X_{n}|^{2}=0. \end{aligned}$$

*Then the numerical solution of Eq*. (13) *is mean*-*square stable*, *where*

$$\begin{aligned} h_{0}=-\frac{-2a_{1}+2a_{2}+2a_{3}\tau+(b_{1}+b_{2}+b_{3}\tau^{2})}{b_{1}(a_{2}+a_{3}\tau )+(b_{2}+b_{3}\tau^{2})(2a_{1}-a_{2}-a_{3}\tau)}. \end{aligned}$$

### Proof

From the second equation of (14), we obtain

$$\begin{aligned} |X_{n+1}|^{2}=\big|X_{n}^{\ast}\big|^{2}+\big|g \bigl(X_{n}^{\ast},X_{n-m},\bar{X}_{n} \bigr)\big|^{2}\triangle W_{n}^{2}+2\bigl\langle X_{n}^{\ast},g\bigl(X_{n}^{\ast},X_{n-m}, \bar{X}_{n}\bigr)\triangle W_{n}\bigr\rangle . \end{aligned}$$

Note that $E(\triangle W_{n})=0$, $E[(\triangle W_{n})^{2}]=h$, and $X_{n}$, $X_{n-m}$, *X̄* are $\mathcal{F}_{t_{n}}$-measurable, hence

$$\begin{gathered} E\bigl\langle X_{n}^{\ast},g\bigl(X_{n}^{\ast},X_{n-m}, \bar{X}_{n}\bigr)\triangle W_{n}\bigr\rangle =0, \\ E\big|g\bigl(X_{n}^{\ast},X_{n-m},\bar{X}_{n} \bigr)\big|^{2}\triangle W_{n}^{2}=\big|g \bigl(X_{n}^{\ast},X_{n-m},\bar{X}_{n} \bigr)\big|^{2}h. \end{gathered}$$

Combining condition (17) and taking expectations on both sides of the above equation,

$$\begin{aligned} E|X_{n+1}|^{2}&\leq E\big|X_{n}^{\ast}\big|^{2}+ \bigl(b_{1}E\big|X_{n}^{\ast}\big|^{2}+b_{2}E|X_{n-m}|^{2}+b_{3}E| \bar{X}_{n}|^{2}\bigr)h \\ &\leq(1+b_{1}h)E\big|X_{n}^{\ast}\big|^{2}+b_{2}hE|X_{n-m}|^{2}+b_{3}hE| \bar{X}_{n}|^{2}. \end{aligned}$$

Next, we should derive the $E|X_{n}^{\ast}|^{2}$ by the first equation of (14),

19 $$ X_{n}^{\ast}-f\bigl(X_{n}^{\ast},X_{n-m}, \bar{X}_{n}\bigr)h=X_{n} . $$

Squaring both sides of Eq. (19), one gets

20 $$ \big|X_{n}^{\ast}\big|^{2}\leq|X_{n}|^{2}+2h \bigl\langle X_{n}^{\ast}f\bigl(X_{n}^{\ast},X_{n-m}, \bar {X}_{n}\bigr)\bigr\rangle . $$

Through the conditions (15), (16), we have

$$\begin{gathered} 2\bigl\langle X_{n}^{\ast}, f\bigl(X_{n}^{\ast},X_{n-m}, \bar{X}_{n}\bigr)\bigr\rangle \\ \quad=2\bigl\langle X_{n}^{\ast}, f\bigl(X_{n}^{\ast},0,0 \bigr)\bigr\rangle +2\bigl\langle X_{n}^{\ast},\bigl(f \bigl(X_{n}^{\ast},X_{n-m},\bar{X}_{n} \bigr)-f\bigl(X_{n}^{\ast},0,0\bigr)\bigr)\bigr\rangle \\ \quad\leq-2a_{1}\big|X_{n}^{\ast}\big|^{2}+a_{2} \bigl(\big|X_{n}^{\ast}\big|^{2}+|X_{n-m}|^{2} \bigr)+2a_{3}\big|X_{n}^{\ast}\bar{X}_{n}\big|. \end{gathered}$$

It is easily to see that for $X_{n}^{\ast}\bar{X}_{n}$ from Sect. 3

$$\begin{aligned} 2X_{n}^{\ast}\bar{X}_{n}=\tau X_{n}^{\ast}+ \frac{h}{2}X_{n-m}^{2}+h\sum _{k=1}^{m-1}X_{n-k}^{2}+ \frac{h}{2}X_{n}^{2}. \end{aligned}$$

Substituting these into Eq. (20) and taking expectations

$$\begin{aligned} E\big|X_{n}^{\ast}\big|^{2}&\leq E|X_{n}|^{2}-2a_{1}hE\big|X_{n}^{\ast}\big|^{2}+a_{2}hE \bigl(\big|X_{n}^{\ast}\big|^{2}+|X_{n-m}|^{2} \bigr) \\ &\quad{}+a_{3}h\Biggl(\tau X_{n}^{\ast}+ \frac{h}{2}X_{n-m}^{2}+h\sum _{k=1}^{m-1}X_{n-k}^{2}+ \frac{h}{2}X_{n}^{2}\Biggr) \\ &\leq(-2a_{1}h+a_{2}h+a_{3}h \tau)E\big|X_{n}^{\ast}\big|^{2}+ E|X_{n}|^{2}+a_{2}hE|X_{n-m}|^{2} \\ &\quad{}+a_{3}h\tau\max_{n-m\leq i\leq n}E|X_{i}|^{2}. \end{aligned}$$

In particular

$$\begin{aligned} E\big|X_{n}^{\ast}\big|^{2}&\leq\frac{1}{1+2a_{1}h-a_{2}h-a_{3}h\tau}E|X_{n}|^{2}+ \frac {a_{2}h}{1+2a_{1}h-a_{2}h-a_{3}h\tau}E|X_{n-m}|^{2} \\ &\quad{}+\frac{a_{3}h\tau}{1+2a_{1}h-a_{2}h-a_{3}h\tau}\max_{n-m\leq i\leq n}E|X_{i}|^{2}. \end{aligned}$$

Hence

$$\begin{aligned} E|X_{n+1}|^{2}&\leq(1+b_{1}h)E\big|X_{n}^{\ast}\big|^{2}+b_{2}hE|X_{n-m}|^{2}+b_{3}hE| \bar {X}_{n}|^{2} \\ &\leq\frac{1+b_{1}h}{1+2a_{1}h-a_{2}h-a_{3}h\tau}E|X_{n}|^{2}+\biggl( \frac {a_{2}h(1+b_{1}h)}{1+2a_{1}h-a_{2}h-a_{3}h\tau}+b_{2}h\biggr) \\ &\quad{}\times E|X_{n-m}|^{2}+\biggl(\frac{a_{3}h\tau(1+b_{1}h)}{1+2a_{1}h-a_{2}h-a_{3}h\tau}+b_{3}h \tau ^{2}\biggr)\max_{n-m\leq i\leq n}E|X_{i}|^{2}. \end{aligned}$$

We can write

$$\begin{aligned} E|X_{n+1}|^{2}\leq PE|X_{n}|^{2}+QE|X_{n-m}|^{2}+R \max_{n-m\leq i\leq n}E|X_{i}|^{2}, \end{aligned}$$

where

$$\begin{gathered} P=\frac{1+b_{1}h}{1+2a_{1}h-a_{2}h-a_{3}h\tau},\qquad Q=\frac {a_{2}h(1+b_{1}h)}{1+2a_{1}h-a_{2}h-a_{3}h\tau}+b_{2}h, \\ R=\frac{a_{3}h\tau(1+b_{1}h)}{1+2a_{1}h-a_{2}h-a_{3}h\tau}+b_{3}h\tau^{2}. \end{gathered}$$

So

$$\begin{aligned} E|X_{n+1}|^{2}\leq(P+Q+R)\Bigl\{ E|X_{n}|^{2},E|X_{n-m}|^{2}, \max_{n-m\leq i\leq n}E|X_{i}|^{2}\Bigr\} . \end{aligned}$$

If $P+Q+R<1$, it is easily to see that $E|X_{n}|^{2}\rightarrow0$ when $n\rightarrow\infty$. By the condition (18), we have

$$\begin{gathered} \bigl[b_{1}(a_{2}+a_{3}\tau)+ \bigl(b_{2}+b_{3}\tau^{2}\bigr) (2a_{1}-a_{2}-a_{3}\tau )\bigr]h^{2}+ \bigl(-2a_{1}+2a_{2}+2a_{3}\tau \\ \quad{}+\bigl(b_{1}+b_{2}+b_{3}\tau^{2} \bigr)\bigr)h< 0. \end{gathered}$$

Namely

$$\begin{gathered} a_{2}h+a_{3}h\tau+b_{1}h+a_{2}b_{1}h^{2}+a_{3}b_{1} \tau h^{2}+b_{3}h \tau^{2}+b_{3}\tau ^{2}(2a_{1}-a_{2}-a_{3} \tau)h^{2} \\ \quad{}+b_{2}h+b_{2}(2a_{1}-a_{2}-a_{3} \tau)h^{2}-2a_{1}h+a_{2}h+a_{3}h\tau< 0. \end{gathered}$$

Therefore, for every step-size $h\in(0,h_{0}]$, $\lim_{n\rightarrow\infty }E|X_{n}|^{2}=0$ holds, the split-step backward Euler method for nonlinear stochastic equations is mean-square stable. The proof is complete. □

## Numerical experiments

In this section, we will discuss the example to verify the theoretical results, considering the following testified equation:

21 $$ \left\{ \textstyle\begin{array}{l} dx(t)=[\alpha x(t)+\beta x(t-1)+\gamma \int _{t-1}^{t}x(s)\,ds]\,dt \\ \phantom{dx(t)=} +[\lambda x(t)+\mu x(t-1)+\eta \int_{t-1}^{t}x(s)\,ds]\,dW(t), \quad t\geq0, \\ x(t)=\xi(t), \quad t\in[-1,0]. \end{array}\displaystyle \right . $$

Taking the parameters $\alpha=-10$, $\beta=2$, $\gamma=1$, $\lambda=0.5$, $\mu =0.2$, $\eta=0.5$, the condition (2) is satisfied.

**Figure 1:**
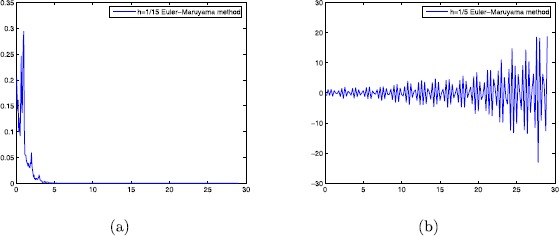
The Euler–Maruyama method with (**a**) $h=1/15$; (**b**) $h=1/5$

*Case 1.* We can easily see that $h_{1}=\frac{12.56}{169}$, $h_{2}=\min\{\frac {1}{10},\frac{12.56}{49}\}$. By Theorem 3.1, $h_{0}=\max\{h_{1},h_{2}\}=\frac{1}{10}$. When the step-size $h\in(0,\frac{1}{10}]$, the Euler–Maruyama method applied to Eq. (21) is mean-square stable. However, the Euler–Maruyama method is unstable when the step-size $h=\frac{1}{5}>\frac{1}{10}$, which is shown in Fig. 1(a) and (b).

**Figure 2:**
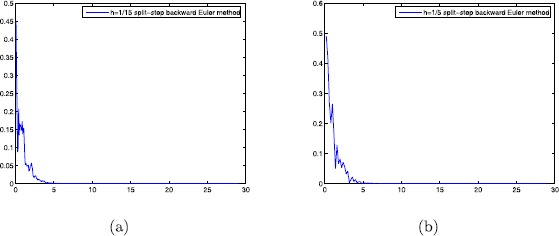
The split-step backward Euler method with (**a**) $h=1/15$; (**b**) $h=1/5$

*Case 2.* We can know $A=[(|\beta\lambda|+|\gamma\lambda|\tau)-\alpha(|\mu |+|\eta|\tau)]^{2}>0$, $\frac{-B+\sqrt{\triangle}}{2A}\approx1.13>1$, and the conditions satisfy Theorem 4.1. Hence for any $0< h<1$, the split-step backward method Euler has general mean-square stability. From Fig. 2, it is easy to confirm general mean-square stability of a numerical solution under the same step-size as Case 1. The results indicate that the split-step backward Euler method achieves superiority over the Euler–Maruyama method in terms of mean-square stability.

*Case 3.* We will address the following nonlinear stochastic delay integro-differential equation:

22 $$ \left\{ \textstyle\begin{array}{l} dx(t)=[-80x(t)+10x(t-1)+10 \int_{t-1}^{t}x(s)\,ds]\,dt \\ \phantom{dx(t)=} +[0.4x(t)+0.4x(t-1)+4 \int_{t-1}^{t}x(s)\,ds]\,dW(t),\quad t\geq0, \\ x(t)=1,\quad t\in[-1,0]. \end{array}\displaystyle \right . $$

It is easy to ascertain that Eq. (22) satisfies the conditions of Lemma 5.1. So

$$\begin{aligned} a_{1}=80,\qquad a_{2}=10,\qquad a_{3}=10,\qquad b_{1}=2,\qquad b_{2}=2, \qquad b_{3}=20, \qquad\tau=1. \end{aligned}$$

Therefore

23 $$ -a_{1}+a_{2}+a_{3}\tau+ \frac{1}{2}\bigl(b_{1}+b_{2}+b_{3} \tau^{2}\bigr)=-96< 0. $$

**Figure 3:**
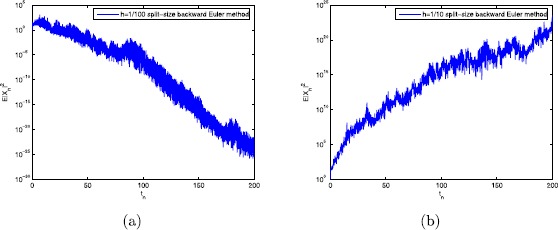
The split-step backward Euler method with (**a**) $h=1/100$; (**b**) $h=1/10$

We should calculate the step-size $h_{0}\approx0.03$ from Theorem 5.1, the data used in all figures are plotted by 200 trajectories. It is proved that the split-step backward Euler method has mean-square stability when $h=0.01$, while *h* dissatisfied $(0,h_{0}]$, that is, $h=0.1>h_{0}$, the split-step backward Euler method is unstable. This is shown in Fig. 3.

## Conclusion

In this paper, we investigate the mean-square stability and general mean-square stability of two numerical methods for a class of linear stochastic delay equations. By comparison, we know that the split-step backward Euler method achieves superiority over the Euler–Maruyama method in terms of mean-square stability. The mean-square stability of numerical method for nonlinear stochastic delay integro-differential equations is eventually confirmed by us.
